# Structural Basis for a Munc13–1 Homodimer to Munc13–1/RIM Heterodimer Switch

**DOI:** 10.1371/journal.pbio.0040192

**Published:** 2006-06-06

**Authors:** Jun Lu, Mischa Machius, Irina Dulubova, Han Dai, Thomas C Südhof, Diana R Tomchick, Josep Rizo

**Affiliations:** **1**Department of Biochemistry, University of Texas Southwestern Medical Center, Dallas, Texas, United States of America; **2**Department of Pharmacology, University of Texas Southwestern Medical Center, Dallas, Texas, United States of America; **3**Department of Molecular Genetics, University of Texas Southwestern Medical Center, Dallas, Texas, United States of America; **4**Center for Basic Neuroscience, University of Texas Southwestern Medical Center, Dallas, Texas, United States of America; **5**Howard Hughes Medical Institute, University of Texas Southwestern Medical Center, Dallas, Texas, United States of America; Princeton UniversityUnited States of America

## Abstract

C _2_ domains are well characterized as Ca ^2+^/phospholipid-binding modules, but little is known about how they mediate protein–protein interactions. In neurons, a Munc13–1 C _2_A-domain/RIM zinc-finger domain (ZF) heterodimer couples synaptic vesicle priming to presynaptic plasticity. We now show that the Munc13–1 C _2_A domain homodimerizes, and that homodimerization competes with Munc13–1/RIM heterodimerization. X-ray diffraction studies guided by nuclear magnetic resonance (NMR) experiments reveal the crystal structures of the Munc13–1 C _2_A-domain homodimer and the Munc13–1 C _2_A-domain/RIM ZF heterodimer at 1.44 Å and 1.78 Å resolution, respectively. The C _2_A domain adopts a β-sandwich structure with a four-stranded concave side that mediates homodimerization, leading to the formation of an eight-stranded β-barrel. In contrast, heterodimerization involves the bottom tip of the C _2_A-domain β-sandwich and a C-terminal α-helical extension, which wrap around the RIM ZF domain. Our results describe the structural basis for a Munc13–1 homodimer–Munc13–1/RIM heterodimer switch that may be crucial for vesicle priming and presynaptic plasticity, uncovering at the same time an unexpected versatility of C _2_ domains as protein–protein interaction modules, and illustrating the power of combining NMR spectroscopy and X-ray crystallography to study protein complexes.

## Abstract 2

In neurons, a heterodimer between Munc13-1 and RIM couples synaptic vesicle priming to presynaptic plasticity. Here, a Munc13-1 homodimer that may regulate this process is identified.

## Introduction

The release of neurotransmitters by Ca ^2+^-evoked synaptic vesicle exocytosis is a central event in interneuronal communication. This process involves a series of steps that include docking of synaptic vesicles to specialized sites of the plasma membrane known as active zones, a priming reaction(s) that leaves the vesicles in a release-ready state, and the actual release of neurotransmitters triggered by Ca ^2+^ influx. These steps are controlled by a complex protein machinery that contains components that have homologs in most types of intracellular membrane fusion such as the SNARE proteins syntaxin, synaptobrevin, and SNAP-25, the Sec1/Munc18 (SM) homolog Munc18–1, and the Rab3 small GTPases, as well as specialized proteins such as synaptotagmin 1, complexins, Munc13s, and α-RIMs (reviewed in [ 1]). Although the mechanism of release is still unclear, clues to this mechanism have emerged from the three-dimensional structures of several complexes of these proteins (reviewed in [ 2]). However, much less is known about protein–protein interactions that couple the basic steps of exocytosis to the regulation of release during presynaptic plasticity processes, which are of critical importance to shape the properties of neural networks and are thought to mediate some forms of information processing in the brain [ 3].

**Figure 1:**
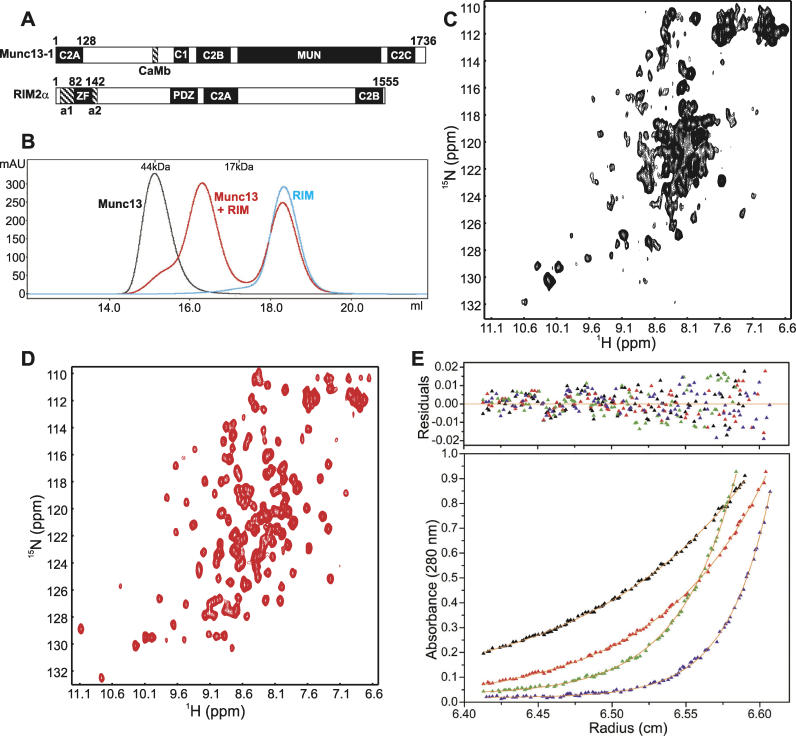
The Munc13–1 C _2_A Domain Homodimerizes (A) Domain structure of Munc13–1 and RIM2α. The calmodulin-binding sequence (CaMb) of Munc13–1 and the helices that flank the RIM2α ZF domain (labeled a1 and a2) are indicated below the diagrams, and residue numbers are indicated above them. (B) Gel filtration analysis of Munc13–1 _3–150_ (black), RIM2α _82–142_ (blue), and the complex between them (red). Elution volumes of two molecular standards are indicated at the top. (C) ^1^H- ^15^N HSQC spectrum of ^15^N-labeled Munc13–1 _3–150_ at 500 MHz. (D) ^1^H- ^15^N HSQC spectrum of ^15^N-labeled Munc13–1 _3–150_ bound to unlabeled RIM2α _82–142_ at 500 MHz. (E) Equilibrium sedimentation analysis of Munc13–1 _3–128_. The data were obtained at centrifugation speeds of 20,000 rpm (black), 25,000 rpm (red), 30,000 rpm (green), and 35,000 rpm (blue). Curves in the bottom panel were generated by fitting the data to a monomer-dimer equilibrium model. The top panel shows the residuals. mAU, milliabsorbance units; ppm, parts per million.

A primary candidate to participate in such coupling is Munc13–1, a large (ca. 200 kDa), multidomain active zone protein (see Figure 1A). Munc13 proteins play a crucial role in synaptic vesicle priming, as shown by the severe disruption of neurotransmitter release caused by *unc13* mutations in invertebrates [ 4, 5], and by the total abrogation of spontaneous, Ca ^2+^-evoked and hypertonic sucrose–induced release observed in double knockout mice lacking Munc13–1 and the closely related isoform Munc13–2 [ 6]. This crucial role in vesicle priming is associated with the C-terminal MUN domain ( Figure 1A), which is sufficient to rescue release in the Munc13–1/2 double knockout mice [ 7]. Other Munc13–1 sequences mediate different presynaptic plasticity processes. Thus, the Munc13–1 C _1_ domain is responsible for diacylglycerol-dependent augmentation of release [ 8], whereas a calmodulin-binding sequence is involved in Ca ^2+^-dependent short-term plasticity [ 9] (see Figure 1A). Moreover, the N-terminal region of Munc13–1 binds to α-RIMs [ 10], which are large (ca. 180 kDa) Rab3 effectors that are localized at active zones and include two closely related isoforms, RIM1α and RIM2α [ 11, 12]. α-RIMs are also required for normal synaptic vesicle priming, and participate in different forms of short- and long-term presynaptic plasticity [ 13– 17]. These forms include mossy-fiber long-term potentiation (LTP), which likely involves a RIM1α/Rab3A interaction as this form of plasticity is abolished in the absence of either RIM1α or Rab3A [ 15, 18]. The severe defects in memory and learning observed in RIM1α knockout mice [ 19] underline the critical importance of these functions.

The physiological significance of Munc13–1/α-RIM binding has been demonstrated by the impairment of vesicle priming observed upon interference with this interaction [ 10, 20], and by the observation of a 60% decrease in Munc13–1 levels in RIM1α knockout mice [ 14]. The Munc13–1/α-RIM interaction requires the C _2_A domain of Munc13–1 and the zinc finger (ZF) domain of α-RIMs [ 20], which is adjacent to the α-helical sequences involved in Rab3 binding (see Figure 1A). This architecture allows formation of a tripartite Rab3/RIM/Munc13–1 complex [ 20]. Hence, in addition to being important for normal synaptic vesicle priming, Munc13–1/α-RIM binding is likely to couple priming to mossy-fiber LTP and perhaps to other forms of RIM-dependent synaptic plasticity. However, the lack of structural information at atomic resolution about this interaction hinders investigation of this hypothesis and elucidation of the coupling mechanism.

Characterizing the Munc13–1/α-RIM interaction is also of general interest for our overall understanding of protein–protein interactions because it involves two different types of widespread protein modules, namely a C _2_ domain and a ZF domain. Whereas it was initially thought that these and other ubiquitous modules (e.g., PDZ, SH2, and SH3 domains) each perform a particular type of activity, it is now clear that many of these modules can participate in diverse interactions. Thus, C _2_ domains perform a variety of functions that often depend on their most common activity, Ca ^2+^-dependent phospholipid binding, but they are also believed to act as protein–protein interaction modules (reviewed in [ 21]). Although the mechanisms of Ca ^2+^/phospholipid binding to C _2_ domains have been extensively characterized [ 22– 24], no high-resolution structures of protein complexes involving C _2_ domains have been described. Similarly, ZF domains exhibit a great functional diversity and have been implicated in multiple protein–protein interactions, but only limited high-resolution information about these interactions is available (reviewed in [ 25]). Hence, understanding the interaction between the Munc13–1 C _2_A domain and the α-RIM ZF domain at atomic detail can provide key insights into the functional diversification of C _2_ domains and ZF domains in general.

Here we show that, in addition to participating in binding to the α-RIM ZF domain, the Munc13–1 C _2_A domain forms a stable homodimer that competes with Munc13–1/α-RIM heterodimerization. Guided by solution nuclear magnetic resonance (NMR) experiments, we have solved the X-ray crystal structure of the Munc13–1 C _2_A-domain homodimer at 1.44 Å resolution, designed a mutation that disrupts homodimerization, and solved the X-ray crystal structure at 1.78 Å resolution of a Munc13–1 fragment bearing this mutation bound to the RIM2α ZF domain. The structures show that the surfaces of the Munc13–1 C _2_A domain involved in homodimerization and heterodimerization are different, but partially overlapping. Our results suggest that a Munc13–1 homodimer–Munc13–1/α-RIM heterodimer switch regulates neurotransmitter release and some forms of presynaptic plasticity, and reveal the structural basis for homo- and heterodimerization. Moreover, our data uncover an unexpected versatility of C _2_ domains as protein–protein interaction modules that underlies this switch, and emphasize that combining NMR spectroscopy with X-ray crystallography provides a powerful approach to investigate protein complexes at atomic resolution.

## Results

### NMR Spectroscopy as a Guide for X-ray Crystallography

NMR spectroscopy and X-ray crystallography provide complementary tools to study protein structure. X-ray crystallography is better suited to elucidate the structures of large proteins and accurately define interfaces of protein complexes, but requires crystallization, which can yield artifacts due to crystal packing. Furthermore, unfolded regions and non-specific aggregation can hinder crystallization, and these properties need to be monitored by alternative techniques. NMR spectroscopy can be performed in solution, and low-resolution information on the conformational and aggregation states of proteins can be quickly obtained even for large species using heteronuclear NMR experiments such as ^1^H- ^15^N heteronuclear single quantum coherence (HSQC). These spectra contain one cross-peak for each non-proline residue of a ^15^N-labeled protein and exhibit well-dispersed cross-peak patterns for well-folded proteins, whereas unfolding or misfolding leads to poor cross-peak dispersion. Moreover, binding interactions and conformational changes can be monitored by perturbations of the cross-peaks, and unusual cross-peak broadening reports on sample aggregation. In this study, we took advantage of the strengths of both techniques, using NMR spectroscopy to optimize protein complexes for crystallization and to obtain structural information in solution that was later employed to interpret the high-resolution structures of these complexes elucidated by X-ray crystallography.

### Homodimerization of the Munc13–1 C _2_A Domain

The predicted C _2_A domain of Munc13–1 encompasses approximately residues 3–130. Using Munc13–1 fragments containing residues 3–132 (Munc13–1 _3–132_), 3–150 (Munc13–1 _3–150_), 3–209 (Munc13–1 _3–209_), and 105–228, we previously showed that the C _2_A domain is essential for binding to the RIM2α ZF domain (RIM2α _82–142_), but additional sequences at its C-terminus are necessary for tight binding [ 20]. In agreement with these conclusions, Munc13–1 _3–150_ and Munc13–1 _3–209_ (but not Munc13–1 _3–132_) largely co-elute with RIM2α _82–142_ in gel filtration experiments with an apparent molecular weight characteristic of a 1:1 heterodimer ( Figure 1B and [ 20]). Interestingly, the apparent molecular weights observed for isolated Munc13–1 _3–132_, Munc13–1 _3–150_, and Munc13–1 _3–209_ in gel filtration were significantly higher than their monomeric molecular weights ( Figure 1B and unpublished data), suggesting that they form stable dimers. These results correlated with the poor quality of the ^1^H- ^15^N HSQC spectrum of isolated ^15^N-labeled Munc13–1 _3–150_, which is inconsistent with a monomeric species ( Figure 1C). In contrast, binding of ^15^N-labeled Munc13–1 _3–150_ to unlabeled RIM2α _82–142_ led to a much higher quality ^1^H- ^15^N HSQC spectrum ( Figure 1D), as expected for a well-behaved 1:1 heterodimer.

We next used analytical ultracentrifugation to determine the oligomerization state of Munc13–1 _3–209_, Munc13–1 _3–150_, and Munc13–1 _3–132_, and of a shorter fragment that we prepared during optimization for our crystallographic studies (Munc13–1 _3–128_; see below). Fitting the data to single ideal species yielded molecular weights of 49,100 Da, 35,200 Da, 30,100 Da, and 26,700 Da, respectively, which are approximately twice the predicted molecular weights of these fragments (24683.4 Da, 17821.3 Da, 16168.4 Da, and 14856.9 Da, respectively). Correspondingly, the best correlations were obtained when the data were fit to an equation describing a monomer/dimer equilibria, yielding dimer dissociation constants of 1.9 nM, 5.2 nM, 80 nM, and 310 nM, respectively (illustrated for Munc13–1 _3–128_ in Figure 1E). Note that dissociation constants below 50–100 nM are not quite accurate under the conditions of our experiments. Nevertheless, these results show that all the N-terminal Munc13–1 fragments studied form stable homodimers and that, although the C-terminal extensions increase the affinity, the C _2_A domain is sufficient and primarily responsible for homodimerization.

### Crystal Structure of the Munc13–1 C _2_A-Domain Homodimer

**Figure 2:**
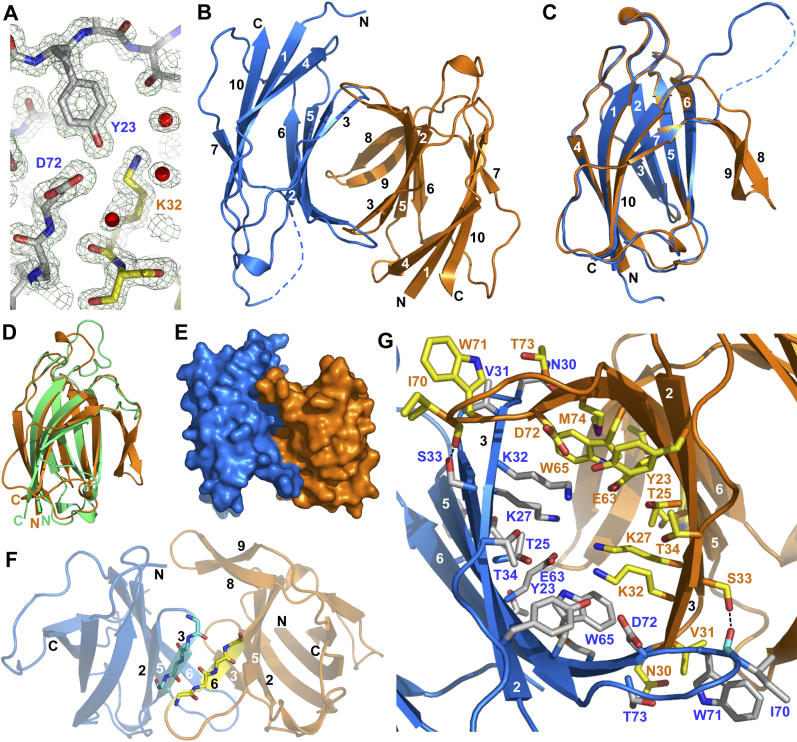
Crystal Structure of the Munc13–1 C _2_A Domain Homodimer (A) A region of the 2F _0_-F _c_ electron density contoured at the 1σ level. (B) Ribbon diagram of the Munc13–1 C _2_A domain homodimer formed by monomers A (blue) and C (orange) showing a top view of the β-barrel like structure. The β-strands are labeled with numbers, and the N- and C-termini are indicated with N and C, respectively. (C) Superposition of monomers A and C. (D) Superposition of monomer C of the Munc13–1 C _2_A domain (orange) and the C _2_ domain of PLC-δ1 (green). (E) Surface representation of the Munc13–1 C _2_A-domain homodimer. (F) Ribbon diagram of the Munc13–1 C _2_A-domain homodimer showing a view perpendicular to that of (B) and illustrating the intermolecular strand–strand interactions that close the β-barrel. The backbone atoms from strand 3 of monomer A and strand 6 of monomer C involved in strand–strand hydrogen bonds are shown as stick models. (G) Close-up view of the dimerization interface. The side chains from residues involved in intermolecular contacts and the Cα carbons of the same residues are shown as stick models, with oxygen atoms in red and nitrogen atoms in blue; Cα carbons are shown with the same color as the ribbon, and other carbons are shown in gray for monomer A and yellow for monomer C. The carbonyl groups of I70, which form hydrogen bonds (dotted lines) with the S33 hydroxyl groups are also shown as stick models. For simplicity, other hydrogen bonds are not shown. All diagrams were generated with Pymol (DeLano Scientific, San Carlos, California).

The ^1^H- ^15^N HSQC spectrum of Munc13–1 _3–132_ ( Figure 1C) is of worse quality than expected for a homodimer, showing that this fragment has a tendency to aggregate. The ^1^H- ^15^N HSQC spectra of Munc13–1 _3–150_ and Munc13–1 _3–209_ ( Figure S1) exhibit further broadening and accumulation of cross-peaks in the center, indicating that the C-terminal extensions are not structured and increase aggregation. Correspondingly, initial screens yielded no crystals for Munc13–1 _3–150_ and Munc13–1 _3–209_, and extensive screens with Munc13–1 _3–132_ only led to needle-like crystal clusters that were not suitable for structure determination ( Figure S1). Intense efforts to obtain well-behaved fragments led to a shorter fragment, Munc13–1 _3–128_, that yielded much higher quality ^1^H- ^15^N HSQC spectra than Munc13–1 _3–132_ ( Figure S1). This fragment readily yielded crystals under 20 different conditions of a basic crystallization screen (Index screen, Hampton Research, Aliso Viejo, California, United States). After condition optimization, we obtained high-quality crystals ( Figure S1) that allowed us to solve the crystal structure of the Munc13–1 C _2_A-domain homodimer at 1.44 Å resolution using molecular replacement. Table 1 describes the structural statistics and Figure 2A shows a representative portion of the electron density.

**Table 1:**
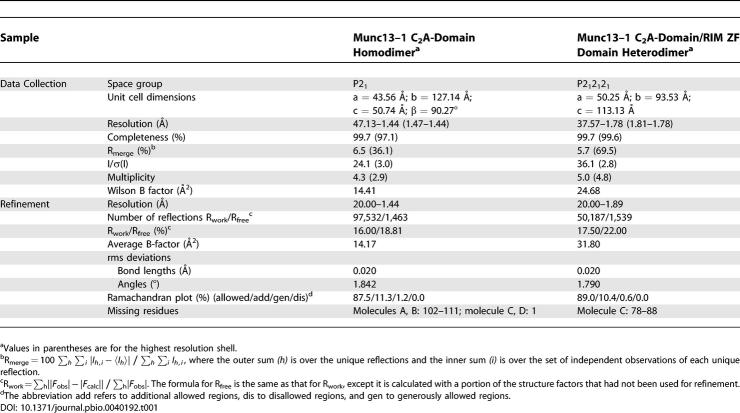
Data Collection and Refinement Statistics

The asymmetric unit of the Munc13–1 C _2_A-domain crystals contains four monomers (referred to as monomers A–D). The monomers can be grouped into two pairs (A/C and B/D, corresponding to one dimer each) based on the extensive buried surface in the interface between the two monomers of each pair (ca. 1,300 Å ^2^). A ribbon diagram of the A/C dimer ( Figure 2B) and a superposition of the two monomers from this dimer ( Figure 2C) reveal that the structures of the two monomers are very similar, but there is a clear difference in the conformation of a long loop that forms a β-hairpin in monomer C (orange) and is partially disordered in monomer A (blue). The structures of corresponding monomers in the two dimers are virtually identical (root mean square [rms] deviation 0.09 Å between all common atoms of monomers A and B, and 0.43 Å between all common atoms of monomers C and D). In contrast, the rms deviation between all common atoms of monomers within a dimer is significantly larger (0.78 Å for A vs. C) due to the different conformation of the β-hairpin loop as well as slight differences in other loops (see Figure 2C).

The Munc13–1 C _2_A domain monomer exhibits a β-sandwich structure formed by two four-stranded β-sheets that is characteristic of C _2_ domains ( Figure 2B and 2C), but the aforementioned β-hairpin formed by a long loop in monomer C (strands 8 and 9) has not been previously observed in other C _2_ domains. A structural comparison using DALI [ 26] showed that, among C _2_ domains deposited in the Protein Data Bank ( http://www.rcsb.org/pdb, the PLC-δ1 C _2_-domain shares the highest structural similarity with the Munc13–1 C _2_A domain (1.65 Å rms deviation for 106 equivalent Cα carbons). The superposition of the PLC-δ1 C _2_ domain and the Munc13–1 C _2_A domain (monomer C) shown in Figure 2D illustrates that the β-sandwich cores of both C _2_ domains are very similar, and that substantial divergence exists in the loops connecting the β-strands, with the most prominent difference being the unusual β-hairpin of the Munc13–1 C _2_A domain.

**Figure 3:**
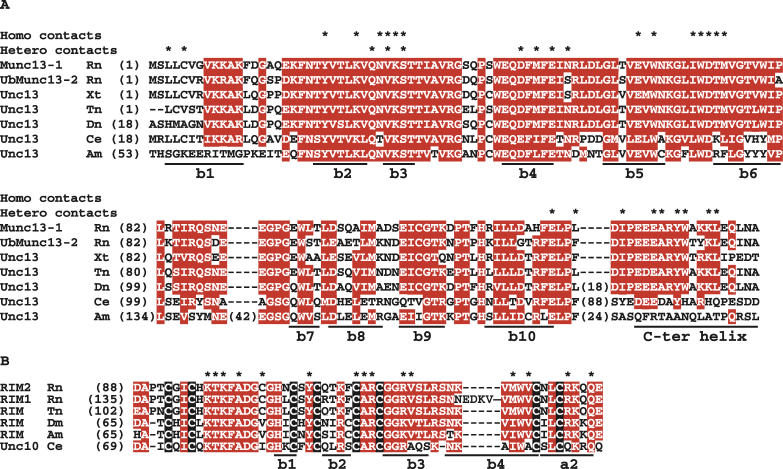
Evolutionary Conservation of Residues Involved in Munc13–1 Homodimerization and Munc13–1/α-RIM Heterodimerization (A) and ( B) Sequence alignments of the Munc13/Unc13 N-terminal sequences including the C _2_A domain and the C-terminal α-helical extension (C-ter) (A), and of the α-RIM/Unc10 ZF domains (B). The eight cysteine residues conserved in all α-RIM/Unc10 ZF domains are shown in white on a black background. Residues conserved in more than 70% of the sequences are highlighted in white with a red background. Residues involved in homo and heterodimerization are indicated by an asterisk (*) above the sequences. The secondary structure elements are indicated below the alignments. Species abbreviations: Am, *Apis mellifera;* Ce, *Caenorhabditis elegans;* Dm, *Drosophila melanogaster;* Dn, *Danio rerio;* Rn, *Rattus norvegicus;* Tn, *Tetraodon nigroviridis;* Xt, *Xenopus tropicalis.*

Dimerization of the Munc13–1 C _2_A domain is mediated by the concave surfaces of the β-sheets formed by strands 3, 2, 5, and 6 of each monomer ( Figure 2B). These surfaces pack in an antiparallel orientation, twisting around each other to maximize intermolecular contacts ( Figure 2B and 2E). This twisted nature of the interface arises from the formation of an eight-stranded β-barrel–like structure by the concave β-sheets of the two monomers, which form intermolecular hydrogen bonds through strands 3 and 6 ( Figure 2B and 2F). In addition to these strand–strand interactions, the interface between the two monomers involves multiple hydrophobic, ionic, and hydrogen-bonding interactions between side chains of both monomers, which are made possible by the complementarity of the hydrophobic and electrostatic properties of the side chains from both monomers that pack against each other ( Figure 2G). Particularly prominent are interactions involving the K32 side chain, which makes extensive hydrophobic contacts with the W65 aromatic ring, forms a hydrogen bond with the Y23 OH group, and interacts electrostatically with the E63 and D72 side chains. Most of the side chains that form the interface between the two monomers are highly conserved through evolution ( Figure 3), suggesting that the ability of the Munc13–1 C _2_A domain to homodimerize is shared in a wide variety of species.

The two monomers of the Munc13–1 C _2_A-domain dimer are largely related by a C _2_ symmetry axis that runs through the center of the β-barrel, but this symmetry is broken by the unusual β-hairpin of monomer C because the same sequence is partially disordered in monomer A ( Figure 2B). The β-hairpin from monomer C appears to form a lid over the β-barrel ( Figure 2B, 2F, and 2G), but its surface is separated from the surface of monomer A by a layer of ordered water molecules and hence does not establish direct intermonomer contacts. However, this β-hairpin is well packed against strand 6 of monomer C itself and appears to be an intrinsic feature of the Munc13–1 C _2_A domain, because the two Munc13–1 monomers present in the crystal structure of the Munc13–1/RIM2 heterodimer also contain this β-hairpin (see below). The observation of this β-hairpin in only one monomer of the Munc13–1 C _2_A-domain homodimer likely arises from steric clashes that would occur if this β-hairpin were formed in both monomers. Interestingly, the ^1^H- ^15^N HSQC spectrum of Munc13–1 _3–128_ ( Figure S1) contains only about 100 backbone cross-peaks. This cross-peak count indicates a symmetry between the two monomers in solution, whereas the absence of approximately 20 cross-peaks in the spectrum suggests that both monomers may alternate in forming the β-hairpin, resulting in a dynamic equilibrium that broadens the cross-peaks from the amide groups of this region.

### Crystallization of a Munc13–1/RIM2α ZF Domain Heterodimer

Munc13–1 _3–150_ is the minimal Munc13–1 fragment for tight binding to the RIM2α ZF domain (apparent K _d_ 0.35 μM); further C-terminal extension to residue 209 provides a moderate increase in binding energy (1.17 kcal/mol based on an apparent K _d_ of 0.07 μM for the Munc13–1 _3–209_ complex) [ 20]. Comparison of the ^1^H- ^15^N HSQC spectra of ^15^N-labeled Munc13–1 _3–150_ or Munc13–1 _3–209_ bound to unlabeled RIM2α _82–142_ ( Figure S2) showed that the C-terminal extension in the latter fragment leads to increased resonance broadening and that the additional cross-peaks arising from this sequence are largely concentrated in the center of the spectrum. These observations suggest that residues 151–209 are mostly unstructured in the Munc13–1 _3–209_/RIM2α _82–142_ complex, which may hinder crystallization. Thus, crystallization trials with the Munc13–1 _3–209_/RIM2α _82–142_ complex failed, although we were also unable to obtain crystals of the Munc13–1 _3–150_/RIM2α _82–142_ complex despite the high quality of its ^1^H- ^15^N HSQC spectrum ( Figure 1D). We reasoned that the presence of small amounts of Munc13–1 _3–150_ homodimer (see Figure 1B) might hinder crystallization of the heterodimer. Hence, we designed two charge-reversal mutations, K32E and E63K, to disrupt Munc13–1 homodimerization based on the crystal structure of the C _2_A-domain homodimer.

**Figure 4:**
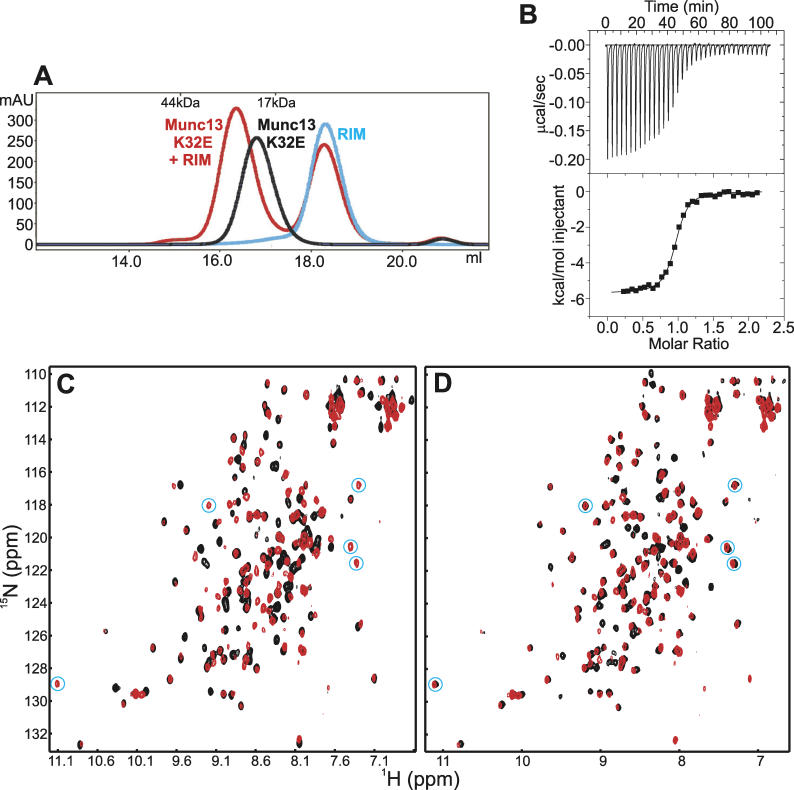
The K32A Mutation Preserves Munc13–1/RIM2α ZF Domain Heterodimerization (A) Gel filtration analysis of Munc13–1 _3–150_(K32E) (black), RIM2α _82–142_ (blue), and the complex between them (red). (B) ITC analysis of the binding of Munc13–1 _3–150_(K32E) to RIM2α _82–142_. (C) ^1^H- ^15^N HSQC spectra of ^15^N-labeled Munc13–1 _3–150_(K32E) alone (black) and bound to unlabeled RIM2α _82–142_ (red) at 800 MHz. (D) ^1^H- ^15^N HSQC spectra of the ^15^N-Munc13–1 _3–150_(K32E)/RIM2α _82–142_ complex (red) and the wild-type ^15^N-Munc13–1 _3–150_/RIM2α _82–142_ complex (black) at 800 MHz. Note that the different appearance of the latter spectrum from that shown in Figure 1D arises from the different magnetic field used. New well-resolved cross-peaks that appear at the edges of the spectrum upon heterodimer formation are circled in blue in (C) and (D). mAU, milliabsorbance units; ppm, parts per million.

Gel filtration showed that both point mutations disrupt dimerization of Munc13–1 _3–128_, Munc13–1 _3–150_, and Munc13–1 _3–209_, but preserve heterodimerization with RIM2α _82–142_ ( Figure 4A and unpublished data). Isothermal titration calorimetry (ITC) confirmed the ability of Munc13–1 _3–150_(K32E) to bind to RIM2α _82–142_ ( Figure 4B), yielding an apparent K _d_ of 0.10 μM and a 1:1 stoichiometry. We attribute this increased apparent affinity compared to the wild-type complex to the lack of competition with homodimerization. The high quality of the ^1^H- ^15^N HSQC spectrum of isolated ^15^N-labeled Munc13–1 _3–150_(K32E) ( Figure 4C, black contours) confirmed its monomeric nature even at 100 μM concentration. Binding to RIM2α _82–142_ induced shifts in numerous cross-peaks of Munc13–1 _3–150_(K32E) ( Figure 4C, red contours), and several new, well-resolved cross-peaks emerged at the edges of the spectrum (circled in blue in Figure 4C). These cross-peaks likely arise from the C-terminal extension of the C _2_A domain (residues 129–150), which may be partially unstructured in isolated Munc13–1 _3–150_(K32E), but is expected to become structured upon binding to the RIM2α _82–142_. Most cross-peaks from the ^1^H- ^15^N HSQC spectrum of the ^15^N-Munc13–1 _3–150_(K32E)/RIM2α _82–142_ complex, including the well-resolved cross-peaks that emerge upon complex formation, coincide with cross-peaks from the wild-type complex ( Figure 4D), showing that the K32E mutation causes little perturbation of the Munc13–1/RIM binding mode. Importantly, the Munc13–1 _3–150_(K32E)/RIM2α _82–142_ complex readily yielded crystals in more than one third of the conditions of a basic crystallization screen (Index screen, Hampton Research) ( Figure S3), whereas crystallization trials with the Munc13–1 _3–209_(K32E)/RIM2α _82–142_ complex failed, as expected from the lack of a defined structure in most of the 151–209 sequence (see above).

### Crystal Structure of the Munc13–1 _3–150_(K32E)/RIM2α ZF Domain Complex

**Figure 5:**
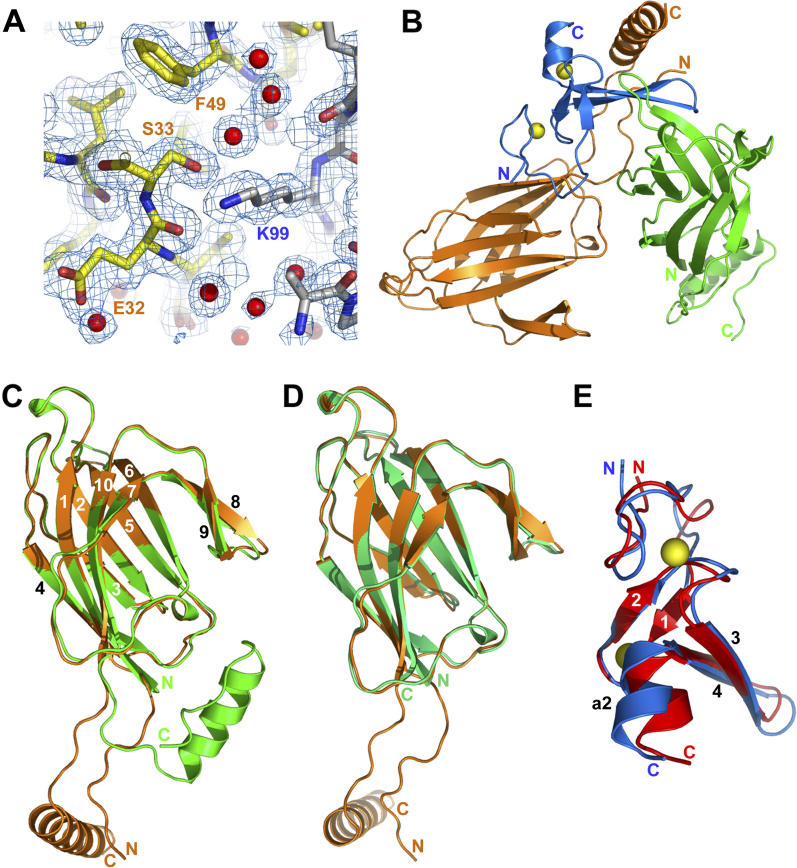
Crystal Structure of the Munc13–1 _3–150_(K32E)/RIM2α ZF Domain Complex (A) A region of the 2F _0_-F _c_ electron density contoured at the 1σ level. (B) Ribbon diagram of the complex with 2:1 stoichiometry seen in the crystals, with RIM2α _82–142_ shown in blue and monomers A and B of Munc13–1 _3–150_(K32E) shown in orange and green, respectively. Zinc atoms are shown as yellow spheres. The N- and C-termini are indicated with N and C, respectively. (C) Superposition of monomers A and B of Munc13–1 _3–150_(K32E) observed in the crystals. The β-strands are labeled with numbers. (D) Superposition of monomer A of Munc13–1 _3–150_(K32E) (orange) with monomer C of the Munc13–1 C _2_A-domain homodimer (green). (E) Superposition of the structure of the RIM2α ZF domain observed in the heterodimer (blue) and its solution structure determined in isolation by NMR spectroscopy (red) [ 20]. Only the zinc atoms from the former structure are shown (yellow spheres). The β-strands are labeled with numbers, and the C-terminal α-helix is labeled a2.

**Figure 6:**
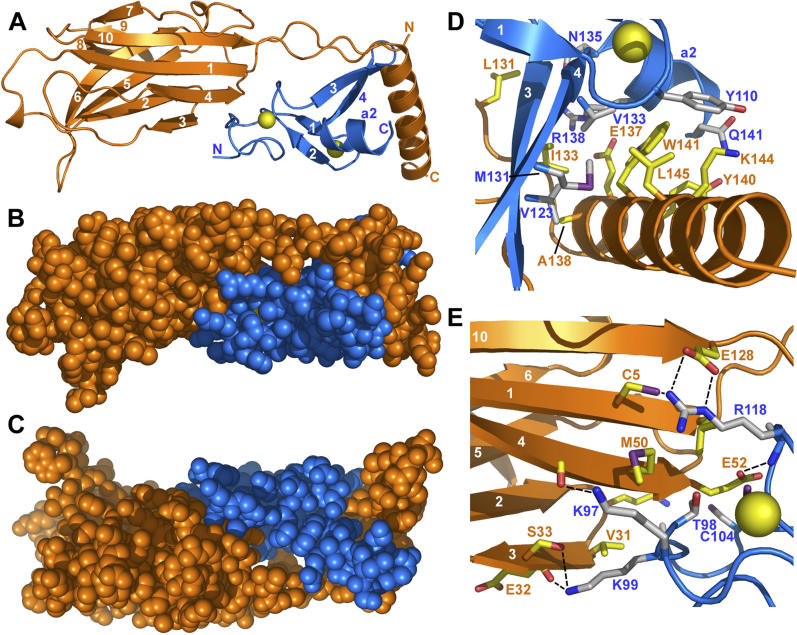
Mode of Munc13–1/RIM2α ZF Domain Binding (A) Ribbon diagram of the complex formed by RIM2α _82–142_ (blue) and monomer A of Munc13–1 _3–150_(K32E) (orange), with the zinc atoms shown as yellow spheres. The β-strands are labeled with numbers, and the α-helix at the C-terminus of the ZF domain is labeled a2. The N- and C-termini are indicated with N and C, respectively. (B) and (C) Space-filling models of the same complex shown in the same orientation as (A) and after rotating approximately 180° around the horizontal axis (C). (D) and (E) Interfaces of the RIM2α ZF domain with the Munc13–1 C-terminal α-helix (D) and C _2_A domain (E). The side chains from residues involved in intermolecular contacts and the Cα carbons of the same residues are shown as stick models, with oxygen atoms in red, nitrogen atoms in blue, and sulfur atoms in purple; Cα carbons are shown with the same color as the ribbon, and other carbons are shown in gray for RIM2α _82–142_ and in yellow for Munc13–1 _3–150_(K32E). Backbone carbonyl groups involved in selected hydrogen bonds (dotted lines) are also shown as stick models. For simplicity, other hydrogen bonds are not shown.

Condition optimization allowed us to determine the structure of the Munc13–1 _3–150_(K32E)/RIM2α _82–142_ complex at 1.78 Å resolution using molecular replacement ( Figures 5 and 6). The structural statistics are described in Table 1, and a representative region of the electron density is shown in Figure 5A. Surprisingly, the asymmetric unit of the crystals contained two molecules of Munc13–1 _3–150_(K32E) (referred to as monomers A and B) and one of RIM2α _82–142_ ( Figure 5B). A superposition of the two Munc13–1 _3–150_(K32E) monomers ( Figure 5C) shows that their C _2_A domains are structurally very similar and include the unusual β-hairpin observed in one of the monomers of the C _2_A-domain homodimer (see above). The C-terminal extension of both Munc13–1 _3–150_(K32E) monomers (residues 129–150) forms an α-helix that is connected to the C _2_A domain by a linker region. In monomer B ( Figure 5B and 5C, green), this α-helix does not contact RIM2α _82–142_ and instead packs against its own C _2_A domain and a crystallographic symmetry mate of monomer A. In contrast, in monomer A ( Figures 5B, 5C, and 6A, orange), the linker region is extended, allowing interactions of the C _2_A domain and the α-helix with opposite sites of the ZF domain. This binding mode nicely explains the requirement of both the C _2_A domain and the C-terminal extension of Munc13–1 for binding to RIM2α _82–142_. In addition, the K97 and K99 side chains of RIM2α _82–142_, which were shown to be critical for Munc13–1 binding [ 20], contact monomer A of Munc13–1 _3–150_(K32E) (see below), but not monomer B. Thus, the binding mode between monomer A and RIM2α _82–142_ observed in the crystals is consistent with all available data obtained in solution, whereas that involving monomer B is not. Moreover, the gel filtration and ITC data ( Figure 4A and 4B), as well as the high quality of the ^1^H- ^15^N HSQC spectrum of Munc13–1 _3–150_(K32E)/RIM2α _82–142_ complex and the observation of only one set of cross-peaks ( Figure 4C), demonstrate a 1:1 stoichiometry. All these results strongly suggest that the monomer A/RIM2α _82–142_ complex observed in the crystals faithfully reflects the true Munc13–1/RIM2α binding mode, whereas the presence of a second Munc13–1 _3–150_(K32E) molecule in the crystals must be considered a consequence of crystal packing.

Superposition of monomer C of the Munc13–1 C _2_A-domain homodimer with monomer A of Munc13–1 _3–150_(K32E) from the heterodimer ( Figure 5D) yielded an rms deviation of 0.52 Å for 127 Cα carbons, showing that binding to RIM2α _82–142_ does not involve large conformational changes in the C _2_A domain. However, the C-terminal sequence of Munc13–1 _3–150_(K32E) likely becomes more structured to form a stable α-helix upon heterodimer formation, based on the ^1^H- ^15^N HSQC spectral changes (see above). The structure of the RIM2α ZF domain in the heterodimer with Munc13–1 _3–150_(K32E) ( Figure 5E, blue) is similar to the solution structure of the isolated RIM2α ZF domain ( Figure 5E, red) [ 20], exhibiting two zinc-binding sites and a central β-hairpin that is flanked on one side by N-terminal loops and on the other side by another β-hairpin and a short C-terminal α-helix (helix a2). Superposition of the two structures yielded an rms deviation of 1.9 Å for 54 Cα carbons, revealing that heterodimer formation induces significant conformational changes in the ZF domain that suggest a substantial malleability and that are consistent with the widespread changes in its ^1^H- ^15^N HSQC spectrum caused by Munc13–1 binding [ 20]. These conformational changes are primarily concentrated at the N-terminal loop region of the ZF domain, which interacts with the Munc13–1 C _2_A-domain β-sandwich, and on the C-terminal region of the ZF domain, where helix a2 and the β-hairpin formed by strands 3 and 4 open up a crevice to interact with the Munc13–1 C-terminal helix ( Figures 5E, 6A, 6D, and 6E).

The two-pronged nature of the Munc13–1/RIM2α interaction, with the primary binding sites for the Munc13–1 C _2_A domain and C-terminal α-helix located on opposite ends of the RIM2α ZF domain, is emphasized by the space-filling models shown in Figure 6B and 6C. Thus, although Munc13–1 _3–150_(K32E) appears to surround a large percentage of the ZF domain surface (the total buried surface area is 2,644 Å ^2^), only the surfaces that contact the Munc13–1 C _2_A domain and the C-terminal α-helix exhibit an overt shape complementarity. In contrast, only one side chain (L131) from the linker region between the C _2_A domain and C-terminal α-helix packs well against the ZF domain. A sequence at the N-terminus of the C _2_A domain arising from the expression vector used also extends across to make contacts with strand 3 of the ZF-domain, but otherwise is not well packed against it.

As shown in Figure 6D, the interaction of the C-terminal α-helix of Munc13–1 _3–150_(K32E) with the crevice between the C-terminal β-hairpin and helix a2 of the ZF domain involves a combination of hydrophobic and polar interactions. On the opposite side of the ZF domain, its N-terminal loop region is rich in basic residues and binds to an acidic region at one tip of the Munc13–1 C _2_A domain β-sandwich through a network of largely polar interactions ( Figure 6E). These interactions include salt bridges as well as backbone–backbone and backbone–side chain hydrogen bonds involving in particular the backbone of strands 3 and 4 of the Munc13–1 C _2_A domain, which form the edges of its two β-sheets on this side of the domain. Particularly prominent are also the multiple interactions involving the K97, K99, and R118 side chains of the ZF domain. This observation explains the abrogation of Munc13–1/RIM binding caused by mutation of K97 and K99 to glutamate [ 20]. Note also that the mutated Munc13–1 side chain (E32) is close to the interface with the ZF domain, but is facing away, consistent with the conclusion drawn from the NMR analysis that the mutation does not substantially alter the binding mode. The alignments in Figure 3 show that most of the Munc13–1 and RIM2α side chains involved in heterodimer formation are highly conserved through evolution, but the Munc13–1 C-terminal helix may not be conserved in invertebrates.

**Figure 7:**
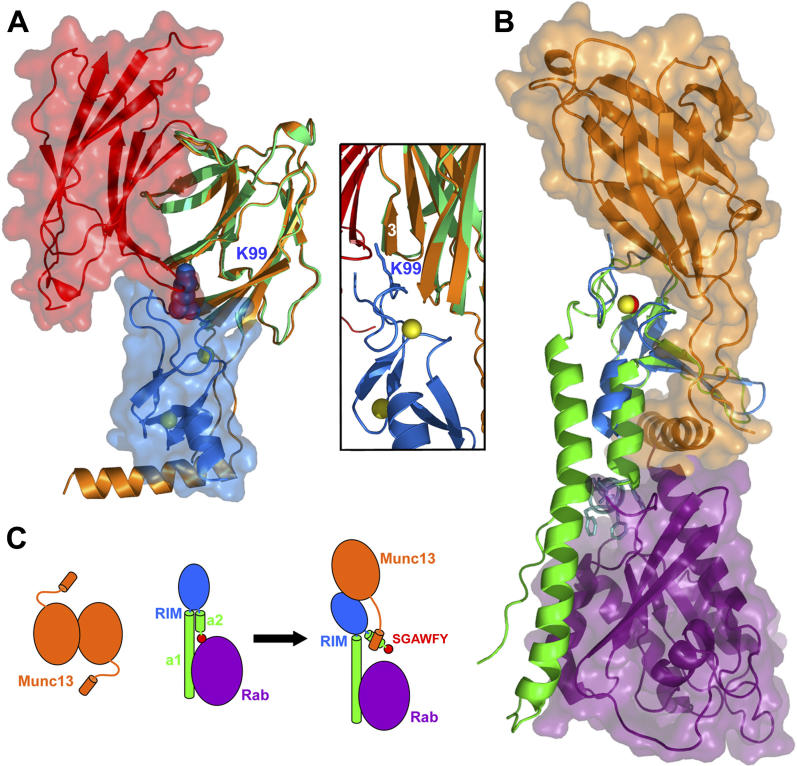
Interplay between the Interactions of Munc13–1, α-RIM, and Rab3s (A) Structures of the Munc13–1 C _2_A-domain homodimer and the Munc13–1 _3–150_(K32E)/RIM2α _82–142_ heterodimer superimposed using monomer C of the homodimer (green) and monomer A of the heterodimer (orange). Ribbon diagrams and transparent surface representations are shown for monomer A of the homodimer (red) and RIM2α _82–142_ (blue) to illustrate the steric clash that would occur if both complexes co-existed. The K99 side chain of the ZF domain is shown as a space-filling model. The inset shows a close-up of the region where this side chain (shown here as stick model) would insert into the homodimer interface (the surface representations are not shown). (B) Structures of the Munc13–1 _3–150_(K32E)/RIM2α _82–142_ heterodimer and the rabphilin/Rab3A complex [ 27] superimposed using the ZF domains of RIM2α (blue) and rabphilin (green). Ribbon diagrams and transparent surface representations are shown for Munc13–1 _3–150_(K32E) (orange) and Rab3A (purple). Zinc atoms are shown as yellow (RIM2α) or red (rabphilin) spheres. The SGAWFF motif of rabphilin is shown as cyan stick model. (C) Model of a cascade of protein–protein interactions that may regulate synaptic vesicle priming and presynaptic plasticity. Munc13–1 is shown in orange and Rab3 in purple. α-RIM is shown in blue (ZF domain), green (helices a1 and a2), and red (SGAWFY motif). The model represents how formation of the tripartite Munc13–1/α-RIM/Rab3 complex involves dissociation of the Munc13–1 homodimer and release of the interaction between the SGAWFY motif and Rab3A.

Only two of the side chains of the Munc13–1 C _2_A domain involved in heterodimerization with RIM2α _82–142_ also participate in homodimerization (V31 and S33; see Figure 3A). Correspondingly, the surfaces of the C _2_A domain involved in homo- and heterodimerization are distinct but contiguous, exhibiting only a small degree of overlap that is sufficient to make both interactions incompatible ( Figure 7A). Particularly noteworthy in this regard are the hydrogen bonds formed by the ZF domain K99 side chain with the S33 hydroxyl group and the backbone carbonyl group of residue 32 of Munc13–1 ( Figure 6E), since these two groups are involved in intermolecular hydrogen bonds in the C _2_A-domain homodimer ( Figure 2F and 2G). Indeed, the RIM2α K99 side chain appears to act like a “dagger” that pierces through one edge of the homodimerization interface to disrupt the homodimer ( Figure 7A).

Our Munc13–1 _3–150_(K32E)/RIM2α _82–142_ structure, together with the crystal structure of a rabphilin/Rab3A complex [ 27], and the sequence homology between rabphilin and RIM2α, also provide an explanation for our previous observations that Munc13–1, RIM2α, and Rab3A form a tripartite complex and that Munc13–1 alters the RIM2α/Rab3A binding mode [ 20]. Thus, NMR studies showed that a long α-helix (helix a1) and an SGAWFY motif at the end of the short α-helix at the C-terminus of the RIM2α ZF domain (helix a2) bind to Rab3A, and that the interaction involving the SGAWFY motif is released upon binding of Munc13–1 to the ZF domain. Figure 7B shows that the α-helical extension at the C-terminus of the Munc13–1 C _2_A domain would have steric clashes with Rab3A upon binding to RIM2α. All these observations lead to the model of Figure 7C, which proposes that formation of the Munc13–1/RIM/Rab3A tripartite complex requires disruption of the Munc13–1 homodimer and a change in the relative orientation of the structural elements of RIM.

## Discussion

Neurotransmitter release constitutes a fundamental means for interneuronal communication and requires tight temporal and spatial regulation. In addition, changes in the efficiency of release in presynaptic plasticity processes are crucial for some forms of information processing in the brain, and presumably arise from modulatory effects on the different steps that lead to release. Although abundant structural information has been obtained for some of the central components of the membrane fusion machinery, little is known about the protein–protein interactions that mediate synaptic vesicle priming and/or couple this key step to presynaptic plasticity processes. The Munc13–1/α-RIM interaction is particularly interesting in this regard since its physiological importance has been demonstrated and both Munc13–1 and α-RIMs are involved in priming and plasticity. Here we uncover a novel interaction that is likely to participate in this coupling, i.e., Munc13–1 C _2_A-domain homodimerization, and describe the three-dimensional structures of the Munc13–1 C _2_A-domain homodimer and a Munc13–1/α-RIM heterodimer, revealing at atomic resolution an unexpected versatility of C _2_ domains as protein–protein interaction modules. Our results illustrate the power of combining X-ray crystallography with NMR spectroscopy to study structural aspects of protein complexes, and suggest that a complex cascade of protein–protein interactions, including a Munc13–1 homodimer–Munc13–1/α-RIM heterodimer switch, may regulate synaptic vesicle priming and some forms of presynaptic plasticity.

### Biological Implications

Munc13s are essential for synaptic vesicle priming [ 6], and this activity resides in their MUN domain [ 7]. At least some of the remaining Munc13 sequences are likely to modulate priming and/or plasticity by regulating MUN domain activity. Disrupting the interaction between the N-terminal region of Munc13–1 and α-RIMs causes a severe impairment in priming [ 10, 20] that is comparable to that observed in RIM1α knockout mice [ 16]. The marked decrease in Munc13–1 levels observed in these mice further supports the physiological importance of the Munc13–1/α-RIM interaction [ 14]. These observations, together with the proposed role of α-RIMs in organizing active zones [ 14], suggest that binding of the Munc13–1 N-terminus to the α-RIM ZF domain may help localize Munc13–1 at the active zone to perform its priming function. Since the Munc13–1/α-RIM interaction competes with Munc13–1 homodimerization, this model predicts that Munc13–1 may exist in solution as a homodimer that needs to be disrupted in order to bind to α-RIMs at the active zone. The physiological role of Munc13–1 homodimerization is currently unclear, but it is plausible that it may play an inhibitory role by hindering α-RIM binding or it may help to stabilize Munc13–1 in the cytoplasm, before engaging in interactions at the active zone. In any case, the observation that only a small overlap exists between the homodimerization and heterodimerization surfaces of the Munc13–1 C _2_A domain ( Figure 7A) suggests that the homodimer may not need to be fully disrupted for α-RIM binding to be initiated. This feature could facilitate a fast transition between the homodimer and the heterodimer during the sequence of events that leads to vesicle priming.

The notion that the Munc13–1/α-RIM interaction, and probably Munc13–1 homodimerization, could couple vesicle priming with presynaptic plasticity emerges naturally from the multiple alterations in short- and long-term plasticity observed in RIM1α knockout mice [ 14– 16]. In particular, the common requirement of RIM1α and Rab3A for mossy-fiber LTP [ 15, 18] suggests that this form of plasticity depends on the RIM1α/Rab3A interaction. Since Rab3A and RIM1α are localized at synaptic vesicles and active zones, respectively, formation of the tripartite Munc13–1/α-RIM/Rab3A complex could help to approximate Munc13–1 to the membrane fusion machinery assembled between the vesicle and plasma membranes [ 20]. The structure of the Munc13–1/RIM2α ZF domain complex described here shows that Munc13–1 binding to the α-RIM/Rab3A complex should lead to a partial steric clash with Rab3A ( Figure 7B), explaining our previous observation that the interaction of the α-RIM SGAWFY motif with Rab3A is released upon Munc13–1 binding [ 20]. The Munc13–1/α-RIM interaction still allows formation of the tripartite complex because helix a1 of α-RIMs is sufficient for Rab3A binding, but is expected to induce a significant reorientation of the N-terminal structural elements of α-RIMs ( Figure 7C). Alternatively, it can be envisaged that Rab3A binding may also cause a rearrangement in α-RIM molecules that were initially bound to Munc13–1. Independently of which event occurs first, the finding that contiguous but partially overlapping surfaces are used by α-RIMs for Munc13–1 and Rab3A binding, and by the Munc13–1 C _2_A domain for homodimerization and heterodimerization, suggests that a synchronized cascade of protein–protein interactions involving Munc13–1, α-RIMs, and Rab3s may control vesicle priming and facilitate fast refilling of the ready-releasable pool of vesicles during repetitive stimulation.

The precise mechanisms linking the interactions between Munc13–1, α-RIMs, and Rab3s to priming and plasticity remain to be determined. It is worth noting that phosphorylation of RIM1α by PKA at an N-terminal site between the ZF and PDZ domains is key for mossy-fiber LTP [ 17], and that the surfaces of Munc13–1 involved in homodimerization and heterodimerization ( Figures 2G, 6D, and 6E) contain several predicted phosphorylation sites (Y23, T25, and Y140). Hence, phosphorylation may be crucial for regulation of inter- and intramolecular interactions of Munc13–1 and α-RIMs during plasticity processes. Evaluation of these possibilities and understanding these mechanisms will require investigation of intramolecular interactions within Munc13–1 and α-RIMs, as well as determination of the interactions involved in the priming activity of the MUN domain, which are currently unclear [ 7]. The three-dimensional structures of the Munc13–1 C _2_A-domain homodimer and the Munc13–1/RIM2α ZF-domain complex described here provide a structural basis for understanding their physiological functions and for designing experiments to further probe these functions.

### C _2_ Domains and ZF Domains as Protein–Protein Interaction Modules

Our results also have implications for the overall understanding of functional diversity in widespread protein modules such as C _2_ domains, PDZ domains, SH2 domains, etc. Each one of these domain families was initially thought to have a specific function common to all members of the family, but exceptions to this notion are constantly emerging. In particular, Ca ^2+^-dependent phospholipid binding is known to be a common activity of many C _2_ domains that is mediated by loops at one tip of the β-sandwich [ 21], but it is now clear that many C _2_ domains do not bind Ca ^2+^ and likely act as protein–protein interaction modules. Our results now reveal for the first time at atomic resolution how a C _2_ domain engages in protein–protein interactions, showing that the Munc13–1 C _2_A domain can in fact establish two different types of interactions that lead to either homodimerization with itself or heterodimerization with the RIM2α ZF domain. The Munc13–1 C _2_A domain does not contain a full complement of Ca ^2+^-binding residues (see [ 28]) and correspondingly does not bind Ca ^2+^ (unpublished data). Interestingly, heterodimerization with the RIM2α ZF domain occurs through one tip of the C _2_A-domain β-sandwich, which is opposite to the tip of the β-sandwich commonly involved in Ca ^2+^/phospholipid binding to C _2_ domains. In contrast, homodimerization involves one side of the β-sandwich. This side contains the concave β-sheet, which interacts with the concave β-sheet of the other monomer in an antiparallel fashion. The interaction involves strand–strand hydrogen bonds through the “naked” edges of the β-sheets, leading to the formation of a β-barrel–like structure. Such form of dimerization is not unusual in β-sheet proteins and may also be a common theme for selected C _2_ domains. Indeed, homodimerization of the piccolo C _2_A domain depends on sequences homologous to strand 3 of the Munc13–1 C _2_A domain [ 29, 30], suggesting that the dimerization mode may be similar. Our demonstration that a C _2_ domain can participate in two types of protein–protein interactions through distinct surfaces, which in turn differ from the usual Ca ^2+^/phospholipid-binding site of C _2_ domains, emphasizes the diversity of interactions that can be mediated by a given protein module. It will thus not be surprising if such versatility is eventually found also in other domain families.

The structural information available on protein–protein interactions involving ZF domains is also limited [ 25]. The X-ray structure of the Munc13–1/RIM2α heterodimer described here provides the first high-resolution view of a complex directly involving a member from the family of ZF domains that includes α-RIMs and other Rab effectors. The structure shows that two surfaces at opposite sides of the RIM2α ZF domain interact with two different structural motifs of Munc13–1 _3–150_. Thus, the N-terminal loop region of the RIM2α ZF domain binds to the tip of the C _2_A-domain β-sandwich, whereas the crevice formed by the C-terminal β-hairpin and helix a2 binds the C-terminal helix of Munc13–1 _3–150_. Interestingly, in the complex between the α subunit of Hif-1 (Hif-1α) and the TAZ1 domain of CREB-binding protein (a ZF domain unrelated to the RIM2α ZF domain), Hif-1α also wraps around the TAZ1 domain, interacting with surfaces at opposite sides of the domain [ 31]. An emerging theme from these observations is that, because of their small size, ZF domains may often use multiple surfaces to increase the affinity and specificity of interactions with target proteins, although further research will be necessary to assess the generality of this notion.

### A Marriage between NMR Spectroscopy and X-ray Crystallography

In the past, NMR spectroscopy and X-ray crystallography were largely viewed as alternative methods for structure determination of biomolecules, but the usefulness of combining the strengths of both techniques is increasingly being recognized [ 32, 33]. On the other hand, two structural genomics studies recently indicated that there is no overt correlation between ^1^H- ^15^N HSQC spectral quality and successful crystallization of protein targets [ 34, 35]. However, it is unclear to what extent the fragment length of each target was optimized in these studies, and a separate structural genomics effort suggested that NMR spectra can be used to identify promising targets for structure determination by X-ray crystallography [ 36]. The data presented here, together with our previous NMR analysis of Munc13–1/α-RIM/Rab3 interactions [ 20], provide a particularly compelling illustration of how NMR spectroscopy can assist in X-ray diffraction studies of protein complexes that present particularly challenging problems for crystallization, and at the same time can provide complementary information. Thus, ^1^H- ^15^N HSQC spectra were instrumental to map the regions involved in these interactions, to identify sequences of the complexes that can hinder crystallization because they are unstructured and promote aggregation, to interpret the X-ray results, and to resolve ambiguities. Altogether, these observations suggest that combining the strengths of NMR spectroscopy in fragment optimization and analysis of protein–protein interactions in solution with the high accuracy of structure determination by X-ray crystallography for biomolecules of any size will be particularly useful to study complex protein networks.

## Materials and Methods

### Protein expression and purification.

Plasmids to express rat Rim2α _82–142_, Munc13–1 _3–132_, Munc13- _3–150_, and Munc13–1 _3–209_ were described elsewhere [ 20]. The construct to express Munc13–1 _3–128_ was generated by PCR and subcloned into a modified pGEX-KG vector [ 37] including a TEV protease cleavage site. The K32E mutation in the Munc13–1 fragments was introduced using the QuickChange Site-Directed Mutagenesis Kit (Stratagene, La Jolla, California, United States) according to the manufacturer's protocol. Unlabeled and isotopically labeled proteins were expressed in bacteria as GST fusions as described [ 20]. The fusion proteins were isolated on glutathione-Sepharose beads (Amersham Biosciences, Little Chalfont, United Kingdom), cleaved from the GST moiety, and further purified by size-exclusion or ion-exchange chromatography. Gel-filtration binding experiments were performed on Superdex S75 or S200 columns (Amersham) in 30 mM Tris-HCl buffer containing 150 mM NaCl, and 1 mM Tris(2-carboxyethyl)-phosphine (TCEP) at pH 7.4.

### Isothermal titration calorimetry.

ITC experiments were performed using a VP-ITC system (MicroCal, Northampton, Massachusetts, United States) at 20 °C in a buffer composed of 30 mM Tris (pH 7.4), 150 mM NaCl, and 1 mM TCEP. The proteins were extensively dialyzed against the buffer, centrifuged, and degassed before the experiment. Typically, 200 μM Rim2α _82–142_ was injected in 35 aliquots of 8 μl into a 1.8-ml sample cell containing 10–20 μM Munc13–1 _3–150_(K32E). Data were fit with a non-linear least-squares routine using a single-site binding model with Origin for ITC v.5.0 (Microcal), varying the stoichiometry *(n),* the enthalpy of the reaction (Δ *H*) and the association constant ( *K*_a_).

### NMR spectroscopy.

^1^H- ^15^N HSQC spectra were acquired at 25 °C on Varian INOVA500, INOVA600, or INOVA800 spectrometers (Varian, Palo Alto, California, United States) using H _2_O/D _2_O 95:5 (v/v) as the solvent. Samples contained 0.1 mM ^15^N-labeled Munc13–1 fragments, alone or together with 0.15 mM unlabeled Rim2α _82–142_, dissolved in 30 mM Tris (pH 7.4), 150 mM NaCl, 1 mM TCEP.

### X-ray crystallography.

The purified Munc13–1 _3–128_ fragment and the Munc13–1 _3–150_(K32E)/Rim2α _82–142_ complex were concentrated to 12 and 10 mg/ml, respectively, in buffer containing 30 mM Tris (pH 7.4), 150 mM NaCl, and 1 mM TCEP. Munc13–1 _3–128_ was crystallized in 0.4 M magnesium formate, 0.1 M sodium acetate (pH 4.5) at 20 °C using the hanging-drop vapor-diffusion method. Crystals appeared overnight and grew to a final size of approximately 0.05 mm × 0.05 mm × 0.35 mm within 3 d. Prior to data collection, crystals were first transferred into 0.02 M sodium acetate buffer (pH 5.5), then transferred step by step to a series of solutions containing 0.02 M sodium acetate (pH 5.5) with increasing concentration of ethylene glycol (5%, 10%, 15%, 20%, 25%, and 30% [v/v] respectively), and finally flash-cooled in liquid propane. Diffraction data were collected at the Structural Biology Center beamline 19ID of the Advanced Photon Source at 100 K to a Bragg spacing ( *d*_min_) of 1.44 Å. The crystals exhibited the symmetry of space group *P*2 _1_, contained four molecules per asymmetric unit, and had unit cell parameters of *a* = 43.56 Å, *b* = 127.14 Å, *c* = 50.74 Å, and β = 90.27°. The Munc13–1 _3–150_(K32E)/Rim2α _82–142_ complex was crystallized in 1.3 M ammonium tartrate (pH 7.0) at 20 °C using the hanging-drop vapor-diffusion method. Crystals appeared overnight and grew to a final size of approximately 0.06 mm × 0.06 mm × 0.25 mm within 4 d. Prior to data collection, crystals were transferred into a solution of 1.4 M ammonium tartrate (pH 7.0) and 20% (v/v) ethylene glycol, and then flash-cooled in liquid propane. Diffraction data were collected at the Structural Biology Center beamline 19ID of the Advanced Photon Source at 100 K to a Bragg spacing ( *d*_min_) of 1.78 Å. The crystals exhibited the symmetry of space group *P*2 _1_2 _1_2 _1_, and had unit cell parameters of *a* = 50.25 Å, *b* = 93.53 Å, *c* = 113.13 Å. Data were processed and scaled in the HKL2000 program suite [ 38].

The Munc13–1 _3–128_ structure was determined via molecular replacement using Phaser [ 39]. Initial model coordinates were obtained by modifying the coordinates of the rat Munc13–1 C _2_B domain (unpublished data). Model building (mainly for the missing loops) was carried out by Arp/warp [ 40] with manual adjustments using O [ 41]. The Munc13–1 _3–150_(K32E)/Rim2α _82–142_ complex was also determined via molecular replacement using Phaser. Initial model coordinates were taken from the structure of Munc13–1 _3–128_. Model building was performed using Arp/warp with minor manual adjustments afterwards. Refinement of the two structures was carried out with Refmac [ 42] of the CCP4 package [ 43] with a random subset of all data set aside for the calculation of *R*_free_. Manual adjustments to the models were carried out with O. After refinement of the protein part was complete, solvent molecules were added using Arp/warp followed by manual adjustments. The models were verified against 2F _o_-F _c_ simulated-annealing omit maps calculated with the program CNS [ 44]. The simulated-annealing omit maps were found to be virtually identical with regular 2F _o_-F _c_ maps. The structures of Munc13–1 _3–128_ and the Munc13–1 _3–150_(K32E)/Rim2α _82–142_ complex have been deposited in the Protein Data Bank.

### Analytical ultracentrifugation.

Sedimentation equilibrium experiments were performed with a Beckman Optima XL-I analytical ultracentrifuge using a 4-position An60Ti rotor and absorbance optical system (Beckman Instruments, Fullerton, California, United States). Each cell has a six-channel carbon-Epon centerpiece with two quartz windows giving an optical path length of 1.2 cm. The sample channels and reference channels were filled with 100-μl proteins and 110-μl buffers, respectively. Absorbance was monitored for each cell in 0.002-cm steps at a wavelength of 280 nm. Samples were centrifuged at 20,000 rpm, 25,000 rpm, 30,000 rpm, and 35,000 rpm at 4 °C until equilibrium had been reached. After equilibrium was reached, overspeed runs at 42,000 rpm were carried out to obtain baseline values of absorbance, which were used in subsequent fits. The loading concentration of Munc13–1 _3–128_, Munc13–1 _3–132_, Munc13–1 _3–150_, and Munc13–1 _3–209_ were 13.52 μM, 13.65 μM, 12.46 μM, and 12.55 μM in 30 mM Tris (pH 8.0), 150 mM NaCl, 1 mM TCEP. The partial specific volumes of Munc13–1 _3–128_, Munc13–1 _3–132_, Munc13–1 _3–150_, and Munc13–1 _3–209_ at 4 °C were calculated from their amino acid composition to be 0.7331 cm ^3^·g ^−1^, 0.7324 cm ^3^·g ^−1^, 0.7327 cm ^3^·g ^−1^, and 0.7170 cm ^3^·g ^−1^, and the calculated monomeric molecular masses are 14,856.9 Da, 16,168.4 Da, 17,821.3 Da, and 24,683.4 Da, respectively. The solvent density was calculated to be 1.005 g·ml ^−1^ at 4 °C. Data sets were fitted to either the single ideal species model or the self-association model using Beckman Optima XL-A/XL-I data analysis software (Origin 6.03). Global analysis was applied to data sets obtained at the different rotor speeds.

## Supporting Information

Figure S1 Analysis of Munc13–1 N-terminal Fragments Using ^1^H- ^15^N HSQC Spectra
(A–D) ^1^H- ^15^N HSQC spectra of Munc13–1 _3–209_, Munc13–1 _3–150_, Munc13–1 _3–132_, and Munc13–1 _3–128._
(E) Example of the needle-like crystal clusters obtained after extensive crystallization screens with Munc13–1 _3–132_.
(F) Example of a crystal obtained for Munc13–1 _3–128_.
(1.3 MB PDF)

Figure S2 Analysis of Munc13–1 _3–150_/RIM2α _82–142_ and Munc13–1 _3–209_/RIM2α _82–142_ Complexes Using ^1^H- ^15^N HSQC Spectra
Superposition of ^1^H- ^15^N HSQC spectra of ^15^N labeled Munc13–1 _3–150_ (black contours) and Munc13–1 _3–209_ (red contours) bound to unlabeled RIM2α _82–142_.
(797 KB PDF)

Figure S3 Gallery of Crystals of the Munc13 _3–150_(K32E)/RIM2α _82–142_ Complex
The crystals were obtained with a basic crystallization screen (Index screen, Hampton Research).
(106 KB PDF)

### Accession Numbers

The structures of the Munc13–1 _3–128_ homodimer and the Munc13–1 _3–150_(K32E)/Rim2α _82–142_ complex have been deposited in the Protein Data Bank ( http://www.rcsb.org/pdb) with accession numbers 2CJT and 2CJS, respectively. The Protein Data Bank accession number for PLC-δ1 is 1DJX.

## Abbreviations

HSQC: heteronuclear single quantum coherence
ITC: isothermal titration calorimetry
LTP: long-term potentiation
NMR: nuclear magnetic resonance
rms: root mean square
SM: Sec1/Munc18
TCEP: Tris(2-carboxyethyl)-phosphine
ZF: zinc finger
