# Supplementary material for: Structural Basis for a Munc13–1 Homodimer to Munc13–1/RIM Heterodimer Switch
Source: PLoS Biol. 2006 Jun 6;4(7):e192. doi: 10.1371/journal.pbio.0040192 (PMC1472246; doi:10.1371/journal.pbio.0040192)
Supplement: Supplementary file 1 [file pbio.0040192.sg001.pdf]

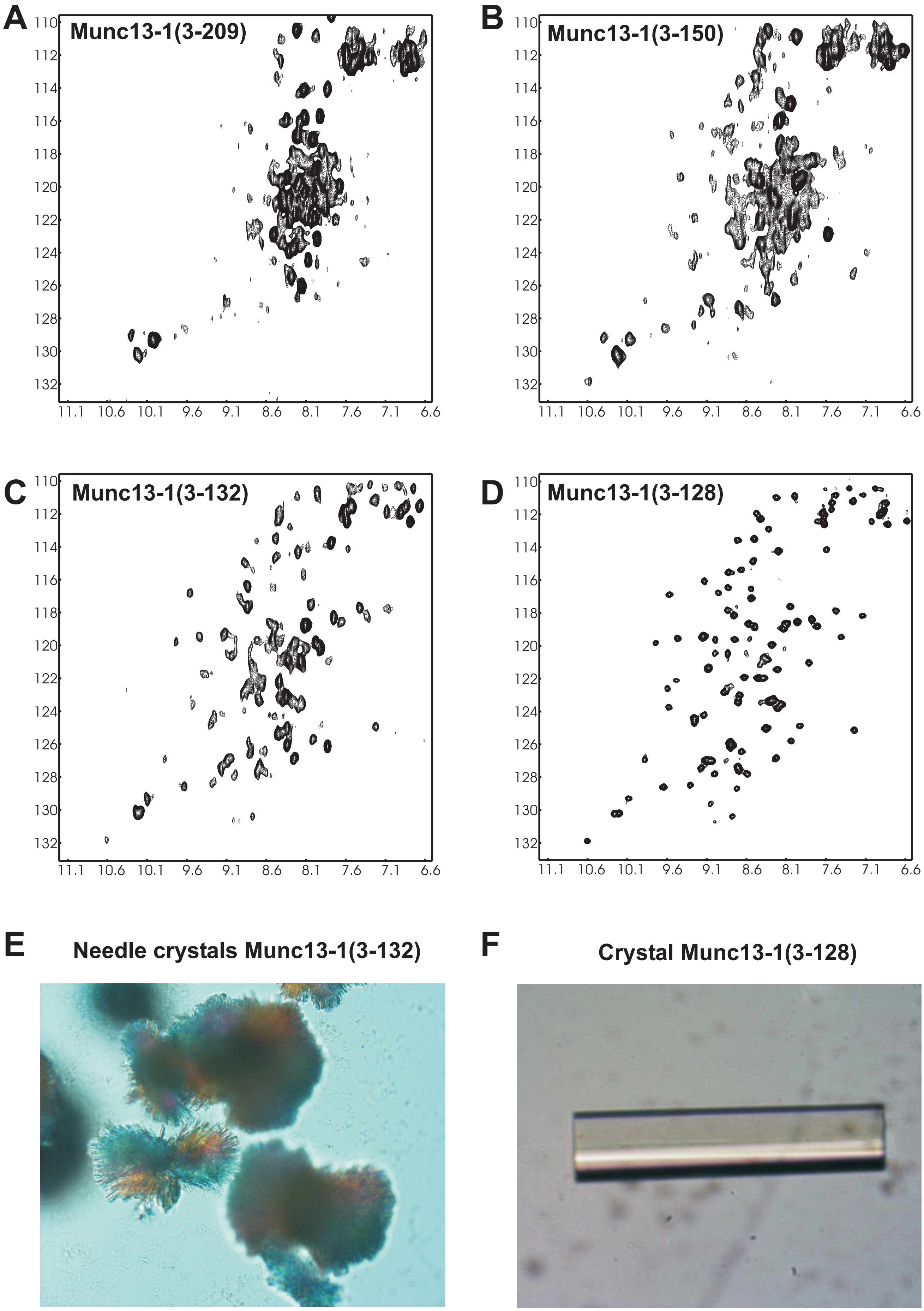

Lu et al., Supplementary Figure 1

Structural Basis for a Munc13-1 Homodimer - Munc13-1/RIM Heterodimer Switch: C_2-domains as Versatile Protein-Protein Interaction Modules

Jun Lu, Mischa Machius, Irina Dulubova, Han Dai, Thomas C. Südhof, Diana Tomchick and Josep Rizo

Supplementary Figure 1. (A-D) ^{1}H-^{15}N HSQC spectra of Munc13-$1_{3\text{-}209}$, Munc13-$1_{3\text{-}150}$, Munc13-$1_{3\text{-}132}$, and Munc13-$1_{3\text{-}128}$. (E) Example of the needle-like crystal clusters obtained after extensive crystallization screens with Munc13-$1_{3\text{-}132}$. (F) Example of a crystal obtained for Munc13-$1_{3\text{-}128}$.
