# Supplementary material for: Structural Basis for a Munc13–1 Homodimer to Munc13–1/RIM Heterodimer Switch
Source: PLoS Biol. 2006 Jun 6;4(7):e192. doi: 10.1371/journal.pbio.0040192 (PMC1472246; doi:10.1371/journal.pbio.0040192)
Supplement: Supplementary file 2 [file pbio.0040192.sg002.pdf]

15N-Munc13-1(3-150)+RIM2α ZF domain

15N-Munc13-1(3-209)+RIM2α ZF domain

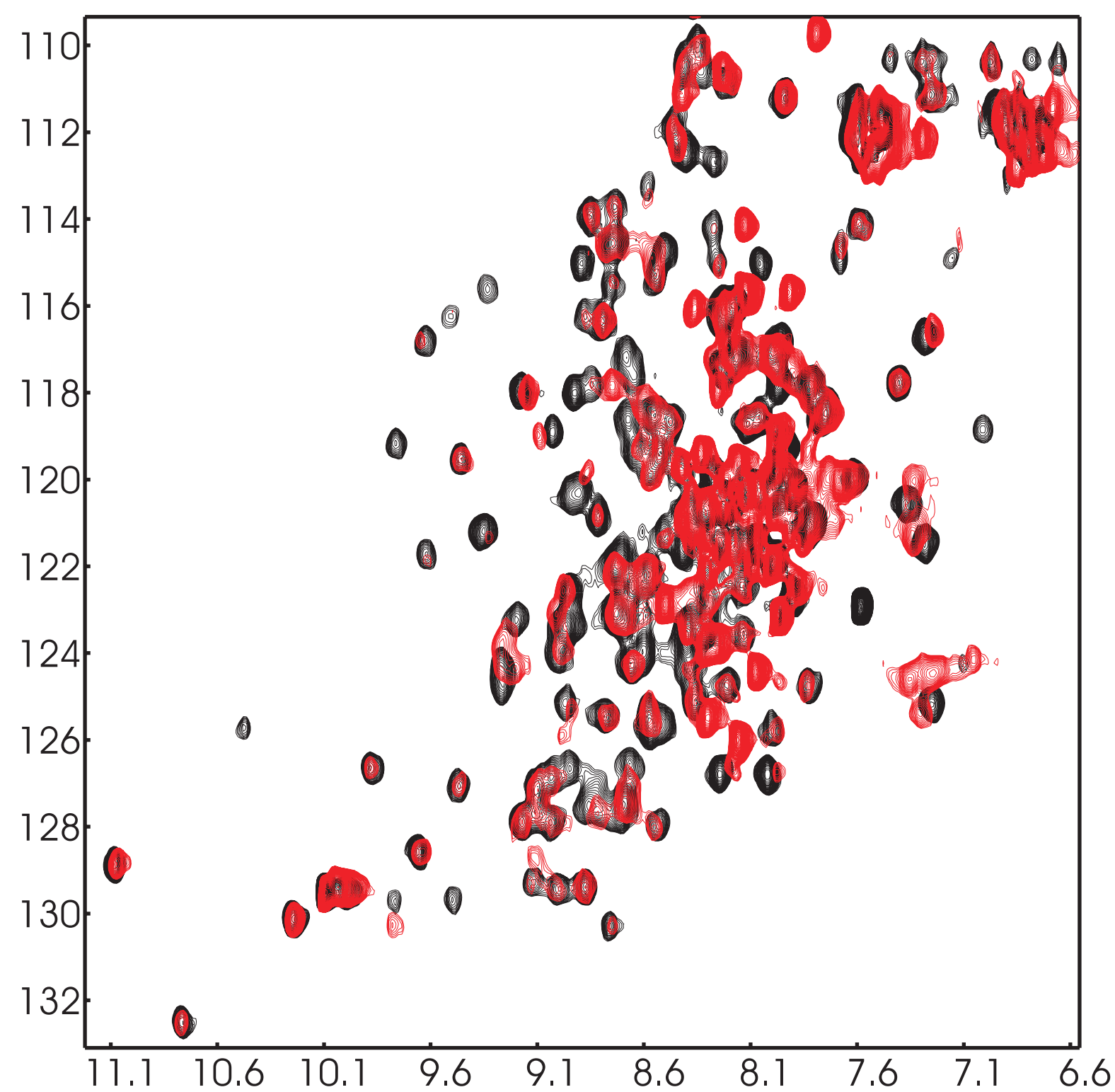

Supplementary Figure 2

Structural Basis for a Munc13-1 Homodimer - Munc13-1/RIM Heterodimer Switch: C_2-domains as Versatile Protein-Protein Interaction Modules

Jun Lu, Mischa Machius, Irina Dulubova, Han Dai, Thomas C. Südhof, Diana Tomchick and Josep Rizo

Supplementary Figure 2. Superposition of ^{1}H-^{15}N HSQC spectra of ^{15}N labeled Munc13-1_{3-150} (black contours) and Munc13-1_{3-209} (red contours) bound to unlabeled RIM2α_{82-142}.
