# Supplementary material for: Structural Basis for a Munc13–1 Homodimer to Munc13–1/RIM Heterodimer Switch
Source: PLoS Biol. 2006 Jun 6;4(7):e192. doi: 10.1371/journal.pbio.0040192 (PMC1472246; doi:10.1371/journal.pbio.0040192)
Supplement: Supplementary file 3 [file pbio.0040192.sg003.pdf]

Crystals of the Munc13-1$_{3\text{-}150}$(K32E)/RIM2$\alpha$ ZF domain complex

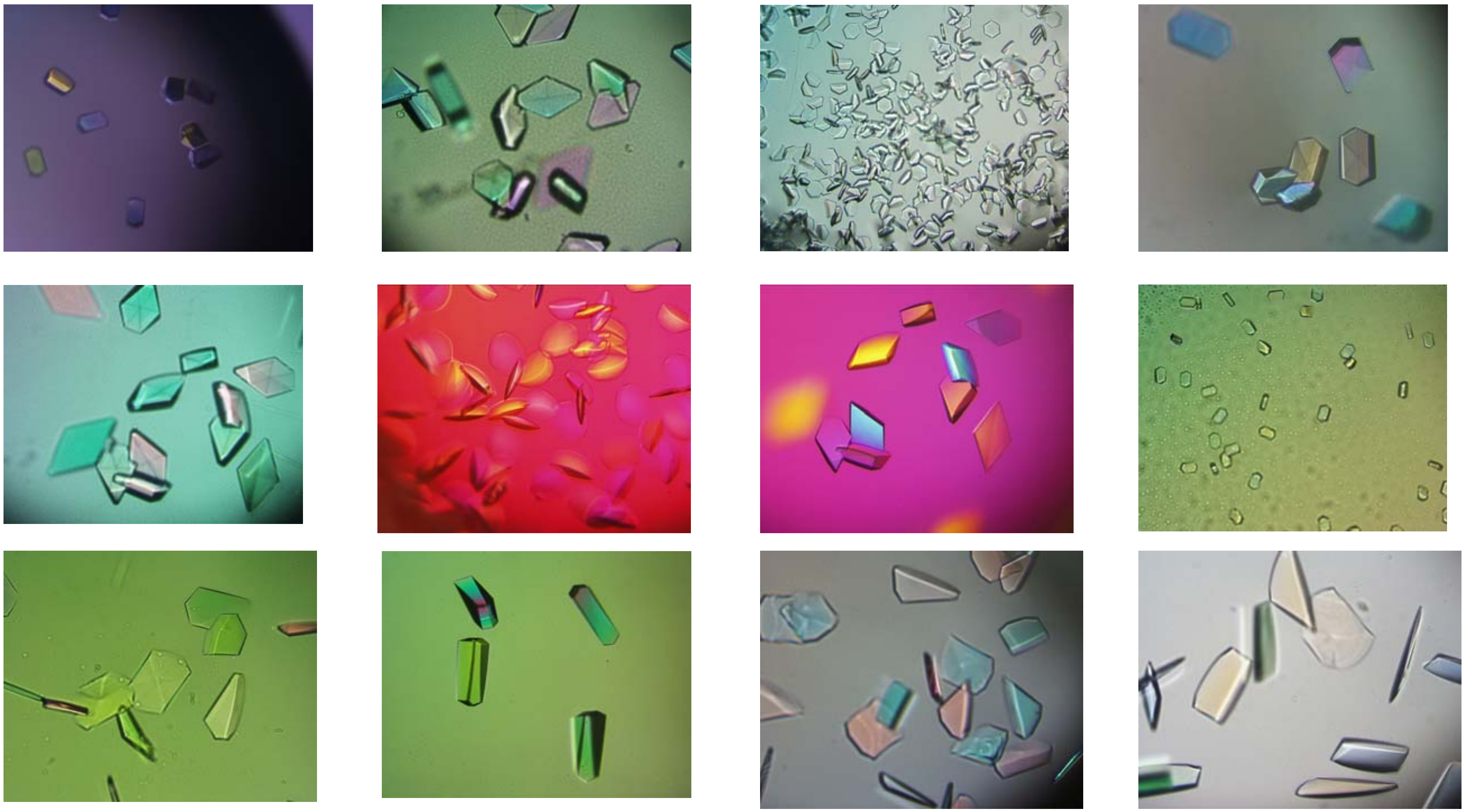

Supplementary Figure 3

**Structural Basis for a Munc13-1 Homodimer - Munc13-1/RIM Heterodimer Switch: $C_2$-domains as Versatile Protein-Protein Interaction Modules**

Jun Lu, Mischa Machius, Irina Dulubova, Han Dai, Thomas C. Südhof, Diana Tomchick and Josep Rizo

**Supplementary Figure 3.** Gallery of crystals obtained with a basic crystallization screen (Index screen, Hampton Research) for the $Munc13_{3\text{-}150}$(K32E)/$RIM2\alpha_{82\text{-}142}$ complex.
